# Emotional Intelligence and Workplace Spirituality in Predicting Career Success of High-Tech Leaders

**DOI:** 10.3390/bs14111009

**Published:** 2024-10-30

**Authors:** Shwu-Ming Wu

**Affiliations:** Department of Human Resource Development, National Kaohsiung University of Science and Technology, Kaohsiung 824004, Taiwan; mingwu@nkust.edu.tw

**Keywords:** emotional intelligence, workplace spirituality, career success, leaders, high-tech industry

## Abstract

In the highly competitive environment of the high-tech industry, leadership roles are often filled with numerous challenges and stressors. Success in the workforce requires a combination of high emotional intelligence and a connection to workplace spirituality. This study aimed to compare demographic differences among high-tech leaders regarding emotional intelligence, workplace spirituality, and career success. It also sought to examine the relationships and effects of emotional intelligence and workplace spirituality on career success, as well as the moderating role of workplace spirituality in these relationships. A survey was conducted with 139 leaders from various high-tech companies in Taiwan. The results showed that male leaders demonstrated higher levels of self-awareness and self-motivation in emotional intelligence, while leaders with higher education levels exhibited greater self-awareness and empathy. Additionally, both emotional intelligence and workplace spirituality were significant predictors of career success. Notably, workplace spirituality moderated the relationship between emotional intelligence and career success. The implications of this study highlight the importance of enhancing emotional intelligence and workplace spirituality to foster leaders’ career success and gain a competitive advantage in the high-tech industry.

## 1. Introduction

Career success has been a significant area of interest for individuals. In the workplace, employees invest efforts and energy to fulfill job requirements and achieve favorable performance, with the potential for leading to career success. Defined as the accomplishment of desirable work-related outcomes resulting from one’s work-related experiences [1], career success has been a crucial focus in management literature. Researchers have shown interest in investigating the determinants and outcomes of career success. This study aims to explore the factors associated with career success.

Emotional intelligence has received increasing empirical attention, reflecting the theoretical proposition that individuals with high emotional intelligence are likely to experience more success in both work-related and non-work aspects of life compared to those with lower emotional intelligence [2]. Goleman (1995, 1998) [3,4] stressed that emotional intelligence is an essential quality for successful business careers. Shabeeba and Manikandan (2019) [5] revealed that dimensions of emotional intelligence, namely emotional management, emotional awareness, socio-emotional awareness, and relationship management, could predict employees’ performance. Through literature reviews, Chagelishvili (2021) [6] addresses emotional intelligence in helping employees in many ways to achieve success at work, and also highlights the need for more attention to the development of employees’ emotional intelligence in organizations. Also, Chrusciel (2006) [7] proposed that emotional intelligence served as a predictor of career success. Thus, this study posits a direct association between emotional intelligence and career success.

Workplace spirituality emerges as a pivotal factor influencing job satisfaction and organizational performance [8] and has the potential to assist individuals in overcoming conflicts in their lives [9], inspiring and fulfilling employees’ work life [10]. Scholars contend that spirituality infuses work with meaning, enhancing employees’ engagement in their tasks and has been shown to have a significant and positive impact on work engagement [11,12] and job satisfaction [13,14,15]. Moitreyee and Lalatendu (2022) [16] further indicated a positive relationship between workplace spirituality and teachers’ professional well-being. From a spiritual framework, de Klerk (2005) [17] explored the construct of meaning in life, highlighting its contribution to improving work-wellness. Consequently, workplace spirituality may serve as an indicator of career success.

Al Ansory, et al. (2022) [18] discovered that spiritual leadership and emotional intelligence positively influenced organizational citizenship behavior and workplace spirituality, while workplace spirituality was identified as a mediator in the relationship between spiritual leadership and organizational citizenship behavior, as well as between emotional intelligence and organizational citizenship behavior. Tischler (2002) [19] also highlighted research indicating a positive correlation between emotional intelligence and workplace success, with spirituality being associated with workplace performance or effectiveness. In the present study, it is anticipated that workers who experience a stronger sense of workplace spirituality congruence will be more engaged in their jobs. Consequently, spirituality at work is likely to have a positive impact on career success. Moreover, emotional intelligence may serve as an indicator of career success. While the predictors of emotional intelligence and workplace spirituality exhibit independent validity, their predictive value may be enhanced when considered together. The potential role of an additional moderator in the relationship between emotional intelligence and career success warrants analysis. Therefore, this study seeks to explore the moderating effect of workplace spirituality on the relationship between emotional intelligence and career success.

Technological advancements, dynamic customer demands, increasing globalization, and growing competition are converging to create organizational environments that are more turbulent and volatile than ever before [20]. Given the uncertain nature of organizational environments, it is not surprising that the human resource development and organizational development literature has increasingly focused on organizational leadership. Leadership is crucial for ensuring effectiveness in organizations and managing change, involving the interaction of executives with other individuals. As emphasized by Joseph et al. (2015) [21], incorporating emotional intelligence competencies into leadership skills is essential for becoming an effective leader. Furthermore, given that leaders today face increased stress and workloads compared to previous years, introducing spirituality into the workplace may contribute to greater career success for leaders. While prior studies have provided valuable insights into how leaders’ characteristics influence career success, limited attention has been given to the role of emotional intelligence and workplace spirituality, two of the most essential qualities in effective leadership. Despite the scarcity of research exploring the connections between emotional intelligence, workplace spirituality, and career success, this study aimed to explore the relationship between emotional intelligence and the career success of leaders in the high-tech industry, with workplace spirituality as a moderating factor. Additionally, it compared the demographic differences of high-tech leaders concerning emotional intelligence, workplace spirituality, and career success.

## 2. Theoretical Framework

### 2.1. Career Success

Career success refers to the accomplishment of desirable work-related outcomes that result from one’s work-related experiences [1]. Career success is the real or perceived achievements that individuals have accumulated as a result of their work experiences. Career success was originally considered from an extrinsic perspective such that objectively measurable achievements, such as pay, promotions, occupational status, and hierarchical level within an organization [22]. Other scholars have argued that subjective career success was an important and distinct facet of career success, which might aid employees in navigating an intricate career environment [23]. Career success is dual-faceted, comprising both objective and subjective dimensions, or extrinsic and intrinsic aspects [24]. The intrinsic or internal dimension of career success, expressed in terms of career and life satisfaction [25], is typically measured through self-perception of career accomplishments and future prospects [22]. Moreover, Heslin (2005) [26] noted an increasing focus on researching both subjective and objective career success. This study delves into both the extrinsic and intrinsic dimensions of career success. Extrinsic career success is gauged by factors such as salary, promotions, and hierarchical position, while intrinsic career success is assessed through career satisfaction.

### 2.2. Emotional Intelligence

The initial model of emotional intelligence in psychology was developed by Salovey and Mayer (1990) [27]. They asserted that emotional intelligence is a subset of social intelligence, involving the ability to monitor one’s and others’ emotions, discriminate among them, and use this information to guide thinking and actions. In contrast, emotional intelligence has been alternatively defined as the ability to adaptively perceive, understand, regulate, and leverage emotions in oneself and others [28]. However, it was Goleman (1995) [3] who popularized the concept of emotional intelligence, originally introduced by Salovey and Mayer (1990) [27]. Goleman (1995, 1998) [3,4], characterized emotional intelligence as the capacity to recognize one’s own feelings and those of others, enhance self-motivation, manage one’s emotions, and effectively handle others’ emotions. Mayer et al. (2004) [29] proposed that emotional intelligence encompasses interrelated functions such as perceiving emotion in oneself and others, using emotion to facilitate decision-making, understanding emotion in oneself and others, and managing emotion in oneself and others. Building on the theoretical model by Salovey and Mayer (1990) [27], Goleman (1995) [3] adopted their original definition and identified emotional intelligence as comprising five major components: self-awareness, managing emotions, self-motivation, and handling relationships. Therefore, in this study, emotional intelligence is measured using these five components.

### 2.3. Workplace Spirituality

Workplace spirituality has been defined in various ways as a value and belief system, a way to get in touch with the inner self, a means for self-realization, and an inner experience [30]. Rego and Cunha (2008) [31] defined workplace spiritually as the recognition that employees have an inner life which nourishes and is nourished by meaningful work taking place in the context of a community. Spirituality at work is about experiencing a sense of purpose and meaning in their work and finding meaning in the performance of tasks [32] as well as experiencing a sense of connectedness to one another and to their workplace community. Initially, spirituality enhances employee well-being and quality of life, subsequently providing a sense of purpose and meaning at work and in task performance [32], along with fostering a sense of interconnectedness and community. Workplace spirituality manifests in employees experiencing a sense of meaning, purpose, community, and transcendence at work [33]. The workplace spirituality conceptualization by Milliman et al. (2003) [15] included the aspects of meaningful work, sense of community, and alignment with organizational values. In this study, following the definition by Rego and Cunha (2008) [31], workplace spirituality refers to an inner life that is nurtured through meaningful work within the context of a community. Specifically, the three key elements, inner life, meaningful work, and a sense of community, outlined in the Spirituality at Work instrument by Ashmos and Duchon (2000) [34], are crucial for measuring workplace spirituality. Therefore, in line with Ashmos and Duchon (2000) [34], workplace spirituality in this study is assessed through these dimensions of inner life, meaningful work, and sense of community.

### 2.4. Demographic Differences of Leaders on Emotional Intelligence, Workplace Spirituality, and Career Success

Petrides and Furnham (2000) [35] reported that males believed they possessed higher emotional intelligence than females. Yousuf and Ahmad’s (2007) [36] study demonstrated emotional intelligence as a mediating variable between the emotional stability trait and managerial effectiveness, as well as between age and job experience. Indartono et al. (2012) [37] explored gender differences in perceptions of workplace spirituality and found that personal factors such as gender, educational background, and family environment have varying effects on the perception of workplace spirituality. Consequently, there exist demographic variations among leaders concerning emotional intelligence and workplace spirituality. Kirchmeyer (1998) [38] observed that women, despite showing lower levels of objective success compared to men, reported equal levels of subjective success. Additionally, age has often been identified as a positive predictor of objective success measures like pay and promotions [39]. Therefore, this study aims to investigate the demographic differences among leaders in terms of emotional intelligence, workplace spirituality, and career success. Thus, Hypothesis 1 suggests:

**Hypothesis** **1:** *There are significant demographic variable differences of leaders on emotional intelligence, workplace spirituality, and career success.*

### 2.5. Emotional Intelligence and Career Success

Emotional intelligence plays a crucial role in the work environment, with employees’ emotional intelligence being able to predict work-related outcomes such as job satisfaction and job performance [40]. Confirming findings from numerous studies, O’Boyle et al. (2011) [41] found that emotional intelligence was highly associated with job performance. Al-Haraisa (2022) [42] identified that emotional intelligence significantly had an impact on career development through organizational socialization. Moreover, Saeedi et al. (2012) [43] confirmed the positive influence of emotional intelligence on career success, highlighting self-awareness as the most impactful aspect on a manager’s career success. Additionally, Chrusciel (2006) [7] suggested that emotional intelligence may serve as a predictor of career success. Thus, Hypothesis 2 suggests:

**Hypothesis** **2:** *Emotional intelligence is positively related to career success.*

### 2.6. Workplace Spirituality and Career Success

Workplace spirituality generally yields a positive impact on job satisfaction [13]. Examining the effects of personality on executive career success in the United States and Europe, Boudreau et al. (2001) [44] discovered that motivational variables are reliably associated with career success. Considering workplace spirituality as reflected in the experience of a sense of meaning and purpose [17], it can be regarded as a motivational variable potentially influencing career success. Notably, Bhaskar and Mishra (2019) [45] found that workplace spirituality enhances career success among employees, suggesting a positive correlation between workplace spirituality and career success. Thus, Hypothesis 3 suggests:

**Hypothesis** **3:** *Workplace spirituality is positively related to career success.*

### 2.7. Moderating Effect of Workplace Spirituality on the Emotional Intelligence and Career Success Relationship

Adawiyah et al. (2011) [46] examined the moderating effects of workplace spirituality on soft total quality management (TQM) and organizational commitment relationships in Islamic banks and found that workplace spiritual values appeared to moderate empowerment and organizational commitment associations. Mousa (2020) [47] discovered that workplace spirituality had an impact on enhancing employee performance. Similarly, Kolodinsky et al. (2008) [48] confirmed that personal spirituality was positively related to intrinsic, extrinsic, and total work rewards satisfaction. Despite the independent validity of emotional intelligence and workplace spirituality predictors, this study suggests that their combined predictive value may increase when considered together. Given the limited empirical research on the interaction effects of emotional intelligence and workplace spirituality on career success, this study postulates that workplace spirituality acts as a moderator in the relationship between emotional intelligence and career success. Thus, Hypothesis 4 suggests:

**Hypothesis** **4:** *Workplace spirituality moderates on the emotional intelligence and career success relationship.*

## 3. Materials and Methods

### 3.1. Research Construct

**Figure 1:**
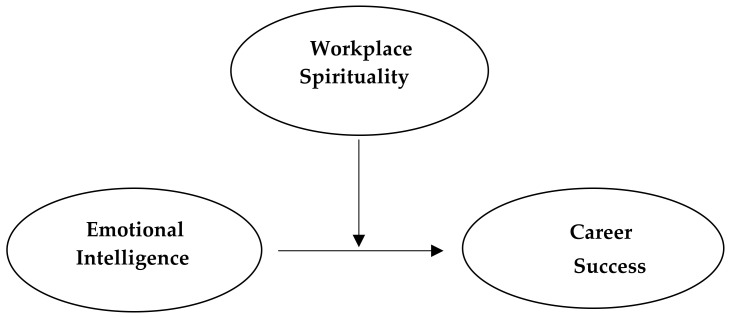
Research Construct.

This study examined the relationships among emotional intelligence, workplace spirituality, and career success, and explored the moderating effect of workplace spirituality on the emotional intelligence and career success relationship. The whole study construct is as Figure 1.

### 3.2. Participants

The participants of this study were 139 managerial leaders, drawn from managerial grades such as executives, managers, or supervisors in Taiwanese high-tech companies. They were recruited from 10 high-tech companies in Taiwan. The 89 men and 50 women ranged in age from 31 to 60 years. All volunteered to respond to the scales. The frequency distribution of demographic variables is presented in Table 1.

### 3.3. Measures

#### 3.3.1. Career Success Scale

The scale was developed based on a literature review and the studies of Greenhaus et al. (1990) [25], employing measurements encompassing both extrinsic and intrinsic dimensions. Extrinsic career success was assessed through salary level, number of promotions, and position in the hierarchy, while intrinsic career success was evaluated by career satisfaction. As all subjects were drawn from managerial leaders, they were considered to have achieved extrinsic career success. In this study, career satisfaction stood as the sole indicator of career success, with responses to the 5 items scored on a 5-point scale. Greenhaus et al. (1990) [25] reported a coefficient alpha of 0.86 for this measure. To assess its applicability in measuring leaders in the Taiwan high-tech industry, two reviewers were invited to revise the items, and a pilot study involving 70 managerial leaders was conducted to test the scale’s reliability and validity. Following an exploratory factor analysis of the 5 items, all items loaded onto one factor with the largest eigenvalues, accounting for 63.56% of the total variance. The Cronbach alpha coefficient of the scale was found to be 0.86.

#### 3.3.2. Emotional Intelligence Scale

Originally developed by Wu (2004) [49] and grounded in the theoretical framework of emotional intelligence proposed by Salovey and Mayer (1990) [27] and Goleman (1995) [3], this scale comprises 25 items that explore five facets of emotional intelligence, self-awareness, managing emotions, self-motivation, empathy, and handling relationships, each represented by 5 items. All item responses were scored on a 5-point scale. Wu (2004) [49] conducted tests for reliability and validity with a sample of 380 teachers and 120 university students, resulting in a Cronbach alpha of 0.81 for the scale. An exploratory factor analysis of the 25 items indicated that those loading on five factors had the largest eigenvalues, explaining 55.2% of the total variance.

To assess the applicability of this scale in measuring leaders in the Taiwan high-tech industry, two reviewers were enlisted to revise the items. A pilot study involving 70 managerial leaders was then conducted, and an exploratory factor analysis of the 25 items revealed that all items, categorized into five factors, had the largest eigenvalues, accounting for 74.92% of the total variance. The Cronbach alpha coefficients for these five subscales ranged from 0.83 to 0.88, with a total scale alpha of 0.93.

#### 3.3.3. Workplace Spirituality Scale

Developed based on the study of Ashmos and Duchon (2000) [34], this scale assesses workplace spirituality through three dimensions: inner life, meaningful work, and sense of community. All 18 items were rated on a 5-point scale. For clarity, a few item wordings underwent revision after scrutiny by two experts. Subsequently, a pilot study involving 70 managerial leaders was conducted to assess the scale’s reliability and validity. Upon performing an exploratory factor analysis of the 18 items, it was found that all items, distributed across three factors (inner life, meaningful work, and sense of community), each comprising 4, 6 and 8 items, respectively, had the largest eigenvalues. These factors collectively accounted for 70.59% of the total variance. The Cronbach alpha coefficients for the three subscales ranged from 0.89 to 0.92, with a total scale alpha of 0.95.

### 3.4. Design and Analyses

The study entailed the development, design and implementation of three psychometric instruments aimed at measuring emotional intelligence, workplace spirituality, and career success. Given that career satisfaction is the sole indicator of career success, all the analyses were conducted based on career satisfaction values. To assess the reliability of the scales, Cronbach’s α coefficients were employed, and principal component analyses were utilized to examine the factor loadings of the scales.

Next, this study compared the demographic (gender, age, education, years of working experience) differences of leaders on emotional intelligence, workplace spirituality, and career satisfaction. A one-way Analysis of Variance (ANOVA) design was formulated to test demographic differences concerning emotional intelligence, workplace spirituality, and career satisfaction. Finally, a predictive correlational design was employed. The correlation matrices among emotional intelligence, workplace spirituality, and career satisfaction were computed. Further, a hierarchical multiple regression analysis was conducted to explore whether emotional intelligence and workplace spirituality served as the best indicators for predicting career satisfaction. Furthermore, the study explored whether workplace spirituality moderated the relationship between emotional intelligence and career satisfaction.

## 4. Results

### 4.1. Demographic Variable Differences of Leaders on Emotional Intelligence, Workplace Spirituality, and Career Success

A one-way ANOVA compared the mean scores of demographic variable (gender, age, education, years of working experience) differences of leaders on the scores of the emotional intelligence, workplace spirituality, and career satisfaction. Only the tests for gender and education variables were significant (*p* < 0.05). Given the significant differences observed only in gender and education variables concerning leaders’ emotional intelligence, means, standard deviations, and *F* tests of gender and education on emotional intelligence of managerial leaders are presented in Table 2. Gender differed significantly on self-awareness and self-motivation, while education showed a significant difference on self-awareness and empathy. Based on means, male leaders scored higher on self-awareness and self-motivation than female leaders. Additionally, the posttest using Scheffé’s analysis revealed that leaders with higher education demonstrated elevated levels of self-awareness and empathy.

### 4.2. Correlations Among Emotional Intelligence, Workplace Spirituality and Career Success

Correlational and hierarchical multiple regression analyses were employed to examine the relationships among emotional intelligence, workplace spirituality, and career success. Table 3 displays the correlations between emotional intelligence and workplace spirituality. Results showed that all variables of emotional intelligence had a statistically significant positive correlation with all variables of workplace spirituality. Using career satisfaction as the indicator for career success, Table 4 displays the correlations of career satisfaction with emotional intelligence and workplace spirituality. The findings revealed significant positive correlations between career satisfaction and all variables of emotional intelligence and workplace spirituality. Consequently, both emotional intelligence and workplace spirituality demonstrated a strong association with career success.

A hierarchical multiple regression analysis was computed to determine whether emotional intelligence and workplace spirituality could predict career satisfaction. The summary of the analysis for predicting career satisfaction is presented in Table 5. The predictors included five dimensions of emotional intelligence and three dimensions of workplace spirituality, with the criterion variable being the total scale of career satisfaction. The findings revealed that managing emotions and handling relationships in emotional intelligence, as well as sense of community in workplace spirituality, were significant predictors of career satisfaction. Therefore, managing emotions, handling relationships and sense of community combined to predict career satisfaction. As shown in Table 4, both emotional intelligence and workplace spirituality exhibited strong associations with career satisfaction. In other words, managerial leaders with higher managing emotions, handling relationships and sense of community corresponded with higher career success.

### 4.3. The Moderating Effect of Workplace Spirituality on the Emotional Intelligence and Career Success Relationship

A hierarchical multiple regression analysis was employed to examine whether workplace spirituality played a moderating role in the relationship between emotional intelligence and career success. The summary of the analysis, predicting the moderating effect of workplace spirituality on the relationship between emotional intelligence and career satisfaction, is presented in Table 6.

Hierarchical multiple regression analysis was utilized to determine whether the interaction of emotional intelligence and workplace spirituality accounted for a significant amount of variance on career satisfaction. At step 1, the independent variable, emotional intelligence, was entered. At step 2, the moderator variable, workplace spirituality, was entered. At step 3, the interaction of emotional intelligence and workplace spirituality was added. As shown in Table 6, the moderation models explained 25% of the variance in emotional intelligence and 37% of the variance in workplace spirituality, and the interaction of emotional intelligence and workplace spirituality combined to explain 40% of the variance on career satisfaction. Although the amount of explained variance attributed to the interaction was somewhat small (*R*^2^ = 0.03), there was still a significant difference on the interaction between emotional intelligence and workplace spirituality (*β* = −2.20, *p* < 0.01). Therefore, the result demonstrated that the interaction of the emotional intelligence and workplace spirituality was significantly related to career satisfaction. Since the interaction *β* is a negative value, workplace spirituality decreased on the emotional intelligence and career satisfaction relationship. So, workplace spirituality played a moderating role in the relationship between emotional intelligence and career success.

**Figure 2:**
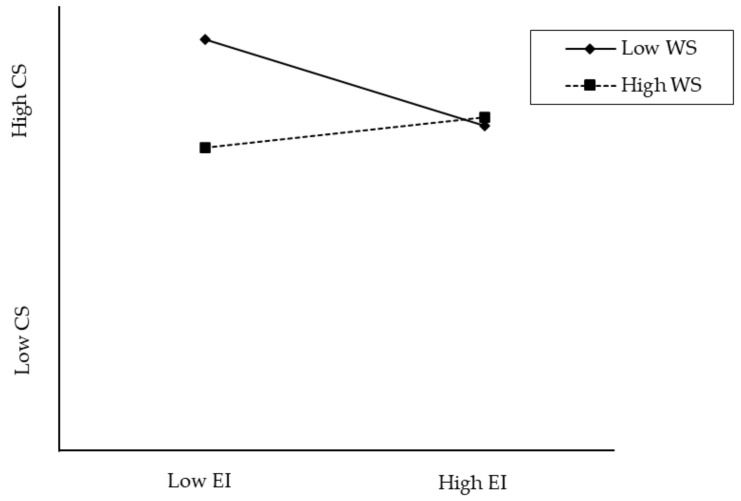
Predicted Regression Lines for Career Satisfaction.

The nature of the interactions can be seen in Figure 2, where career satisfaction was regressed on the emotional intelligence variable for different degrees of workplace spirituality. As shown in Figure 2, low emotional intelligence predicted career satisfaction better where there was high workplace spirituality. In low workplace spirituality, managerial leaders with high emotional intelligence performed higher in career success than those high in workplace spirituality.

## 5. Discussion

Following the development of measures for emotional intelligence, workplace spirituality and career success among leaders in the high-tech industry, this study conducted a comparison of demographic differences among managerial leaders on these three variables. Subsequently, the study aimed to explore the relationships and effects of emotional intelligence and workplace spirituality on career success. The reliability and validity tests for the measures of emotional intelligence, workplace spirituality and career success indicated that these scales possessed mostly adequate properties. Then, some important conclusions were drawn from the present findings. Male leaders demonstrated higher self-awareness and self-motivation, while leaders with higher education showed higher self-awareness and empathy. Additionally, strong positive relationships were observed between emotional intelligence and workplace spirituality with career success. Both emotional intelligence and workplace spirituality was found to be highly associated with career success and emerged as the best predictors of career success. Notably, workplace spirituality played a moderating role in the relationship between emotional intelligence and career success.

Emotional intelligence involves self-awareness, which helps individuals gain a deep understanding of how they react to their emotions and how these reactions impact others [3]. Self-awareness is the capacity to recognize and identify one’s feelings, while self-motivation involves directing emotions toward a goal through emotional self-control, delayed gratification and impulse restraint. Individuals with a high degree of emotional intelligence are aware of what they are feeling, what their emotions mean, and how these emotions affect others. As individuals progress to higher positions or leadership roles, they tend to develop greater self-awareness and self-motivation, particularly among male leaders. This observation aligns with the findings of Petrides and Furnham (2000) [35], which suggest that males generally exhibit higher emotional intelligence. Additionally, leaders with higher educational levels tend to demonstrate increased awareness and empathy, which are crucial for career advancement within an organization. In particular, emotional intelligence is considered essential for achieving career success.

Studies has shown that emotional intelligence (EQ) is a better predictor of success than intelligence quotient (IQ) [3]. Individuals with higher emotional intelligence experience greater job satisfaction, are more productive, and find more opportunities for advancement [40]. The strong association between emotional intelligence and career success supports the findings of Saeedi et al. (2012) [43], confirming that emotional intelligence positively influences career success. This result is in line with the proposition by Chrusciel (2006) [7], which suggests that emotional intelligence serves as a predictor of career success.

Workplace spirituality also displayed a positive correlation with career success, supporting the findings of Bhaskar and Mishra (2019) [45], which suggest that workplace spirituality enhances career success among employees. Consistent with prior studies [13,15], workplace spirituality generally has a positive impact on job satisfaction, thereby extending its positive influence to career success. Notably, Lalatendu (2021) [50] found that both emotional intelligence and workplace spirituality are positively linked to employee performance. Leaders who exhibit high emotional intelligence and a strong sense of workplace spirituality contribute to improved workplace performance, which in turn promotes their career success.

As expected, workplace spirituality was confirmed as a moderator in the relationship between emotional intelligence and career success. However, the moderation effect was found to attenuate the relationship between emotional intelligence and career success. Specifically, having high workplace spirituality, managerial leaders with low emotional intelligence exhibited higher career success. Conversely, having low workplace spirituality, leaders with high emotional intelligence demonstrated higher career success. This finding suggests that leaders who prioritize workplace spirituality may not necessarily require higher emotional intelligence to achieve career success. This aligns with the findings of Mousa (2020) [47], who established that workplace spirituality positively influences employee performance. Additionally, a study by Kolodinsky et al. (2008) [48] supported the notion that personal spirituality is positively correlated with intrinsic, extrinsic and overall work reward satisfaction.

## 6. Conclusions and Implications

The measures employed to assess emotional intelligence, workplace spirituality and career success demonstrated reliability and validity, making them effective tools for examining leaders in the Taiwan high-tech industry. Notably, male leaders exhibited higher self-awareness and self-motivation in emotional intelligence compared to their female counterparts, while leaders with higher education demonstrated greater self-awareness and empathy than those with lower education levels. The implications for female leaders emphasize the need to enhance self-awareness and self-motivation, while leaders with lower educational levels should focus on elevating self-awareness and empathy to enhance their professional growth.

Both emotional intelligence and workplace spirituality emerged as strong indicators of career success. Consequently, the implication drawn from the finding is that leaders should prioritize enhancing emotional intelligence and workplace spirituality, thereby promoting career success and fostering a competitive advantage in the high-tech industry. Workplace spirituality was found to play a moderating role in the relationship between emotional intelligence and career success. An additional implication of this finding is that encouraging workplace spirituality can not only promote leaders’ career success but also enhance overall work performance.

## 7. Contributions and Limitations

Spiritual leadership is an emerging paradigm within the broader context of workplace spirituality. It is grounded in a higher purpose and intrinsic values, aiming to motivate individuals through meaning and purpose. This leadership style focuses on inspiring people to find deeper satisfaction in both their work and life [17]. Spiritual leadership offers numerous benefits to both employees and organizations, including enhanced employee well-being, increased morale, motivation, job satisfaction, commitment and improved job performance [51]. A workplace culture rooted in spiritual leadership also encourages positive interactions, empathy and compassion among employees, creating a more supportive and cohesive work environment. This, in turn, leads to improved teamwork and collaboration [51]. In contrast, non-spiritual leadership primarily focuses on organizational goals, profitability and efficiency. It emphasizes the achievement of tangible, measurable outcomes and relies on extrinsic motivation. This leadership style tends to be more pragmatic in its approach to decision-making and leadership [52]. Therefore, to make a practical contribution, leaders who operate with a profound sense of purpose and guide their employees with values such as love and compassion are more likely to enhance employee performance and contribute to individual career success.

Emotional intelligence is essential for effective leadership, as it involves the ability to recognize, understand and manage both one’s own emotions and the emotions of others. Workplace spirituality focuses on fostering a sense of purpose, belonging and connection within the work environment. Leaders face ongoing challenges and stress. Emotional intelligence helps leaders manage stress, while workplace spirituality provides a sense of purpose and inner strength, enabling them to remain resilient and focused on long-term goals. By integrating emotional intelligence and workplace spirituality, leaders are better equipped to navigate the complexities of leadership while cultivating a workplace that is both emotionally supportive and spiritually fulfilling. Moreover, this holistic approach serves as a practical contribution, enabling leaders to achieve greater career success.

The high-tech industry in Taiwan is known for its rapid growth, innovation and competitive nature. Taiwanese culture emphasizes hard work as a key to achieving career success. Leaders in this field often face high levels of stress and must perform well, making emotional intelligence and workplace spirituality even more important. In fast-paced, high-pressure environments, leaders in Taiwan’s high-tech industry must manage both their personal stress and team morale. High emotional intelligence enables leaders to handle stress effectively, stay composed, and provide emotional support to their teams, which is essential for maintaining productivity and achieving career success in a demanding industry. Workplace spirituality can offer leaders inner strength and resilience, helping them stay focused and grounded despite the challenges of the high-tech industry. Leaders who find meaning and purpose in their work are better equipped to navigate the pressures of the industry, which can lead to greater career success, especially in environments like the high-tech industry, where adaptability, resilience and strong relationships are key drivers of performance.

While the study focused on a sample of 139 managerial leaders, it is recommended that future study considers extending the sample size to enhance external validity. Furthermore, the scales utilized in this study, offering psychometrically sound, self-reporting measures of emotional intelligence, workplace spirituality and career satisfaction, can serve as valuable resources for subsequent researchers. Future investigations may also explore alternative methods for assessing reliability and validity to contribute to a more comprehensive understanding of the relationships examined.

## Figures and Tables

**Table 1:** Frequency Distribution of Demographic Variables.

Demographic Variables	Categories	Frequencies	%
Gender	1. Male	90	64.7
	2. Female	49	35.3
Age	1. Below 30	26	18.7
	2. 31–40	61	43.9
	3. 41–50	40	28.8
	4. 51–60	12	8.6
Education	1. Below College	15	10.8
	2. Bachelor	93	66.9
	3. Master and Above	31	22.3
Years of Working	1. 1–3	44	31.7
	2. 3–5	47	33.8
	3. 5–7	23	16.5
	4. More than 7	25	18.0

Note. N = 139.

**Table 2:** Means, Standard Deviation, and Univariate F Tests of Gender and Education on Emotional Intelligence of Managerial Leaders.

Variables	Demographic Variables	M	SD	F	Scheff’e
Self-awareness	Gender				
	1. Male	20.56	2.13	17.51 **	
	2. Female	18.88	2.44		
Self-motivation	1. Male	19.51	2.49	4.37 **	
	2. Female	18.59	2.44		
Self-awareness	Education				
	1. Below College	20.07	3.10	3.52 *	3 > 2
	2. Bachelor	19.62	2.36		
	3. Master	20.90	1.76		
Empathy	1. Below College	19.60	2.94	3.96 *	3 > 2
	2. Bachelor	18.94	2.37		
	3. Master and Above	20.35	2.43		

Note. N = 139. * *p* < 0.05 ** *p* < 0.01.

**Table 3:** Correlations between Emotional Intelligence and Workplace Spirituality.

Variables	Sense of Community	Meaningful Work	Inner Life	Total Scale of Workplace Spirituality
Self-awareness	0.41 **	0.42 **	0.49 **	0.48 **
Managing Emotions	0.60 **	0.55 **	0.46 **	0.62 **
Self-motivation	0.55 **	0.54 **	0.61 **	0.63 **
Empathy	0.55 **	0.46 **	0.54 **	0.58 **
Handling Relationships	0.59 **	0.56 **	0.56 **	0.64 **
Total Scale of Emotional Intelligence	0.71 **	0.67 **	0.70 **	0.78 **

Note. N = 139. ** *p* < 0.01.

**Table 4:** Correlations of Career Satisfaction with Emotional Intelligence and Workplace Spirituality.

Variables	Career Satisfaction
Self-awareness	0.22 **
Managing Emotions	0.52 **
Self-motivation	0.29 **
Empathy	0.33 **
Handling Relationships	0.51 **
Total Scale of Emotional Intelligence	0.50 **
Sense of Community	0.62 **
Meaningful Work	0.53 **
Inner Life	0.41 **
Total Scale of Workplace Spirituality	0.61 **

Note. N = 139. ** *p* < 0.01.

**Table 5:** Summary of Hierarchical Multiple Regression for Emotional Intelligence and Workplace Spirituality Predicting Career Satisfaction.

Criterion Variable	Predictor Variables	Beta	Multiple R	Multiple R^2^	t
Career Satisfaction	Managing Emotions	0.20	0.52	0.27	2.41 *
	Handling Relationships	0.27	0.60	0.35	2.64 **
	Sense of Community	0.42	0.67	0.44	3.98 **

Note. N = 139. Multiple R and Multiple R^2^ (cumulative values). * *p* < 0.05 ** *p* < 0.01.

**Table 6:** Summary of Hierarchical Multiple Regression for The Moderating Effect of Workplace Spirituality on the Emotional Intelligence and Career Satisfaction Relationship.

Steps	Predicator Variables	Criterion variable: Career Satisfaction		
		Model 1 (*β*)	Model 2 (*β*)	Model 3 (*β*)
Step 1 Independent	Emotional Intelligence	0.50 **	0.08	1.07 **
Step 2 Moderator	Workplace Spirituality		0.55 **	1.88 **
Step 3 Interaction	Emotional Intelligence x Workplace Spirituality			−2.20 **
R		0.50	0.61	0.63
R^2^		0.25	0.37	0.40
ΔR^2^		0.25	0.12	0.03
F		45.94 **	40.01 **	30.24 **

Note. Multiple R and Multiple R^2^ (cumulative values) ΔR^2^ (incremental). ** *p* < 0.01.

## Data Availability

The data are available upon request.
